# Supplementary material for: Evaluating the Antitumor Potential of Cannabichromene, Cannabigerol, and Related Compounds from Cannabis sativa and Piper nigrum Against Malignant Glioma: An In Silico to In Vitro Approach
Source: Int J Mol Sci. 2025 Jun 13;26(12):5688. doi: 10.3390/ijms26125688 (PMC12192948; doi:10.3390/ijms26125688)
Supplement: Supplementary file 1 [file ijms-26-05688-s001.zip › ijms-3417299-supplementary.pdf]

Supplementary material

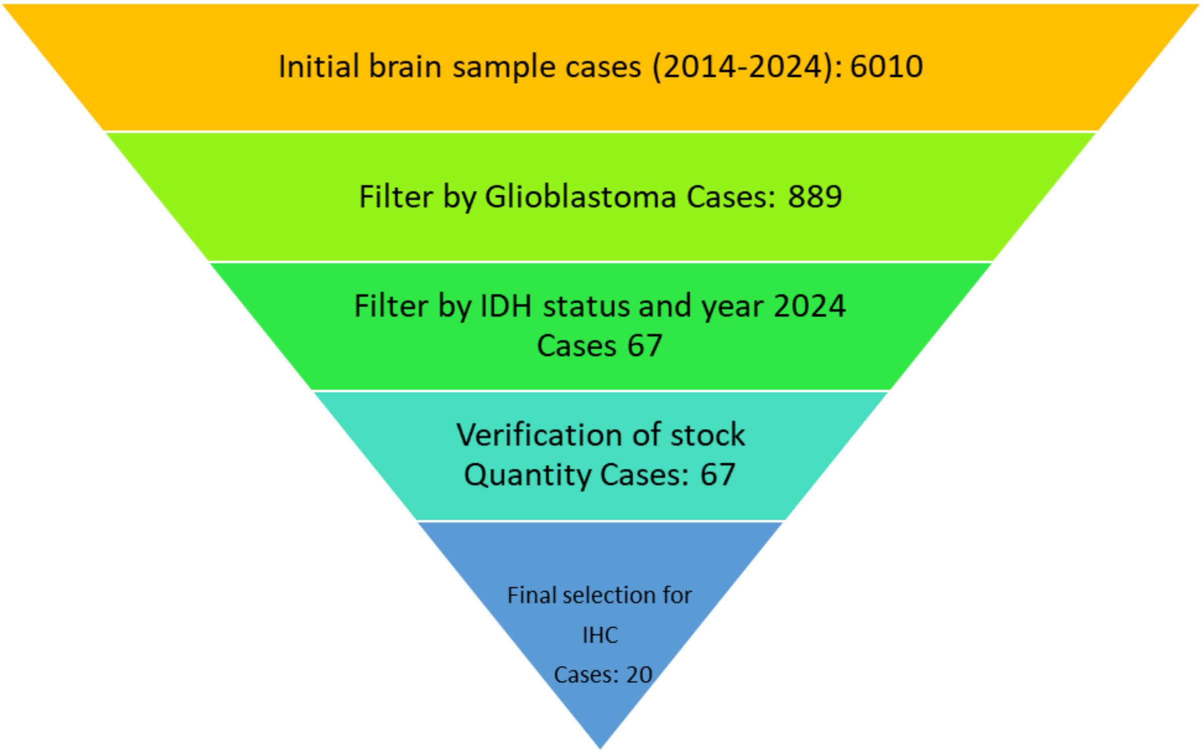

Figure S1. Brain samples procedure.

Table S1. Anatomical and Molecular Features of Selected Patients with Available Tumor Samples for Immunohistochemistry Assays in colombian patients.

Patient Code	Age	Gender	Anatomical Location	GFAP	OLIG2	ATRX	KI67 (%)	P53
24B47049	40	M	Left Frontoparietal-Temporal	YES	YES	YES	30	NO MUT
23B057041	48	F	Right Temporal	YES	YES	YES	20	MUT
24B44594	53	F	Left Parieto-Occipital	YES	YES	YES	60	NO MUT
24B032463	56	M	Unspecified Temporal	YES	YES	YES	70	NO MUT
23B120763	57	F	Supratentorial (Unspecified Level)	YES	YES	NO	30	NO MUT

23B071982	57	F	Left Parietal	YES	YES	YES	30	NO MUT
23B070613	57	F	Right Periventricular	YES	YES	YES	60	NO MUT
23B083227	57	F	Left Parietal	YES	NA	NA	50	NO MUT
23B058359	58	M	Left Frontoparietal	YES	YES	YES	40	NO MUT
24B075538	58	M	Right Temporoparietal	YES	NA	YES	10	MUT
23B083770	58	F	Left Frontoparietal	YES	YES	YES	40	NO MUT
23B058359	58	M	Left Frontoparietal	YES	YES	NA	40	NO MUT
23B052549	66	F	Supratentorial Temporal	YES	YES	YES	30	NO MUT
23B060465.2	68	M	Left Parietal	YES	YES	YES	40	MUT
23B060465.1	68	M	Left Parietal	YES	YES	YES	40	MUT
24B063506	68	M	Right Parieto-Occipital	YES	NA	YES	30	NO MUT
24B073483	73	M	Right Parietal	YES	NA		60	MUT
23B071585	76	M	Supratentorial Midline	YES	YES	YES	40	MUT
23B69783	77	F	Left Frontal	YES	YES	YES	30	NO MUT
24B053954	78	M	Left Frontoincisural	YES	YES	YES	30	NO MUT

COLSA24 DRNA	CONTROL	CONTROL	Control Cortex (Non-tumoral)	CONTROL	CONTROL	CONTROL	CONTROL	CONTROL

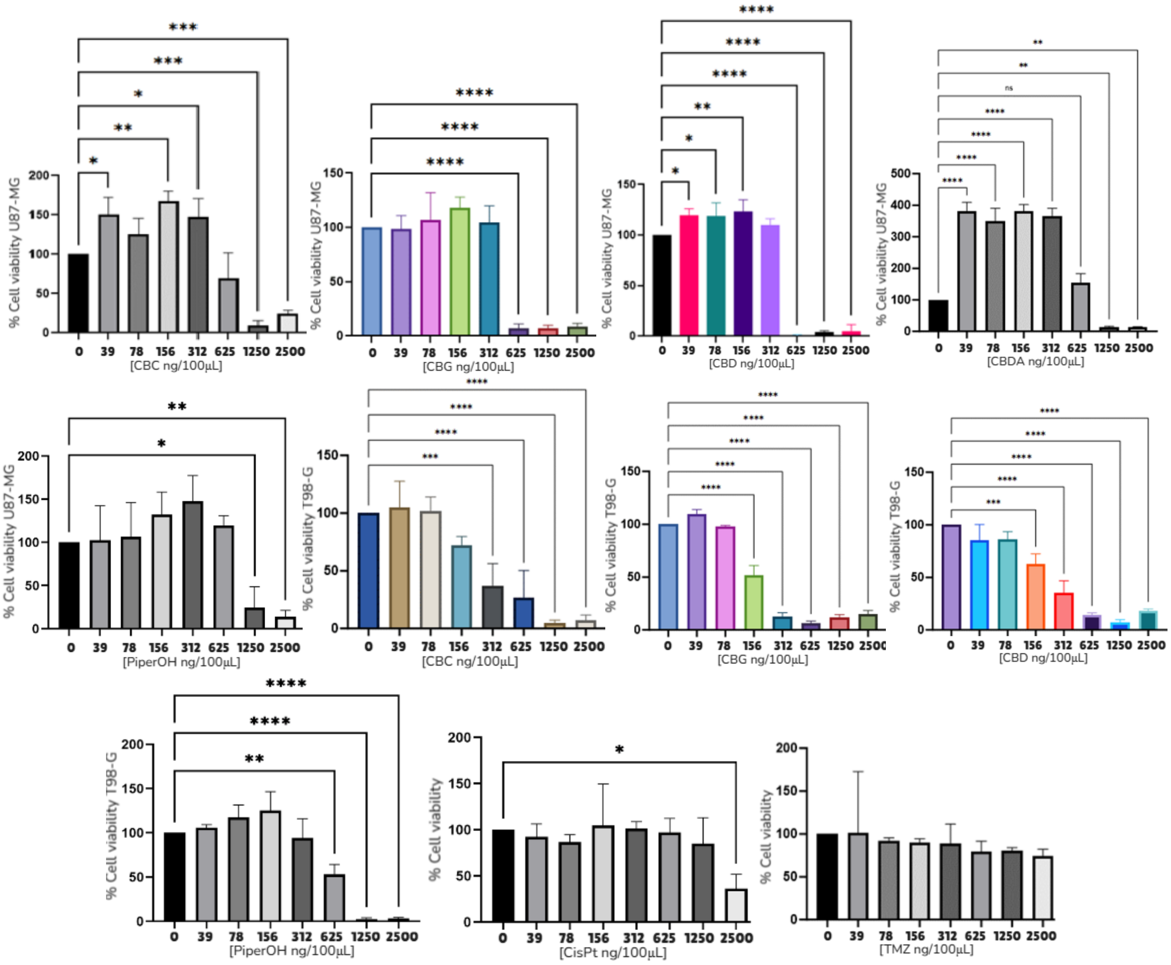

Figure S2. MTT viability assays of U87MG and T98G, 24h.

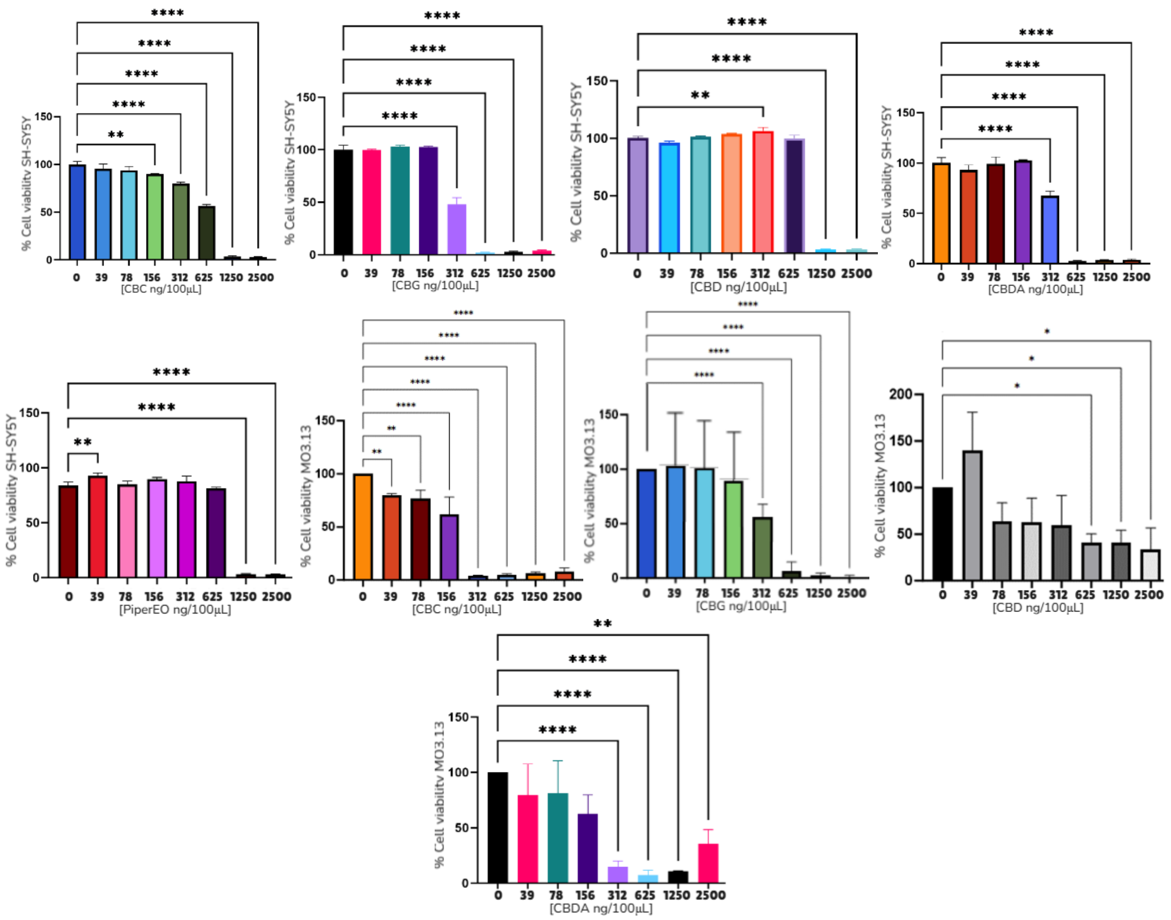

Figure S3. MTT viability assays of SHSY-5Y and MO3.13.

(a)

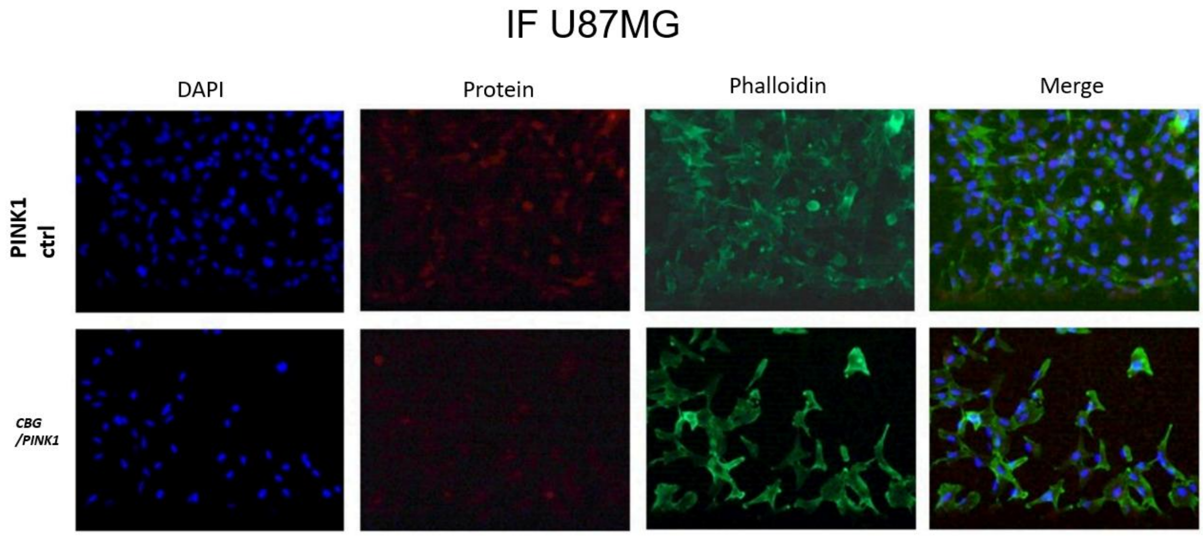
IF U87MG
DAPI
Protein
Phalloidin
Merge
PINK1 ctrl
CBG /PINK1

(b)

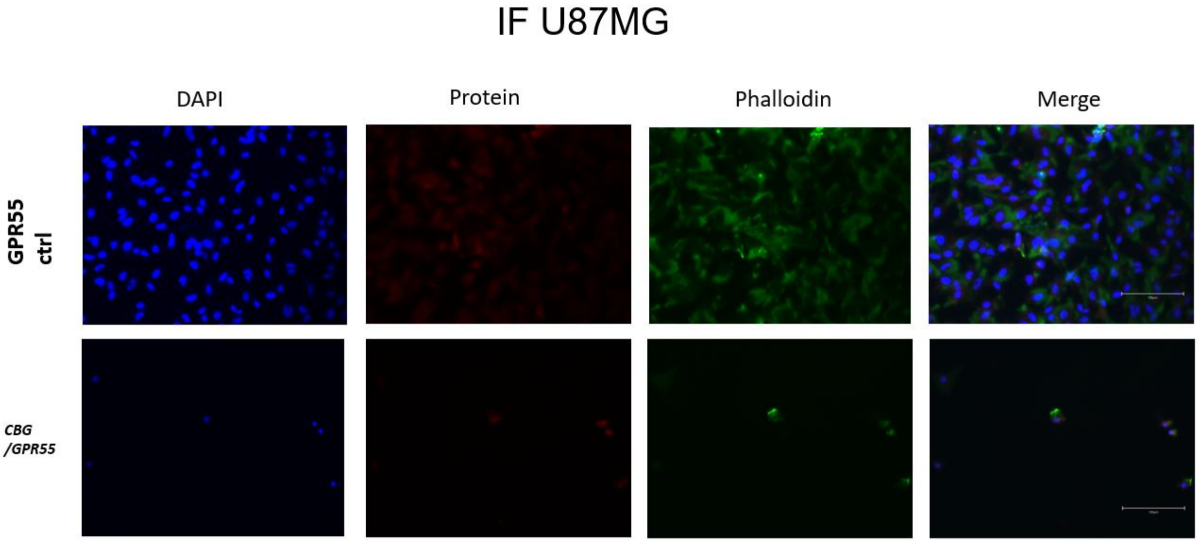
IF U87MG
DAPI
Protein
Phalloidin
Merge
GPR55 ctrl
CBG /GPR55

(c)

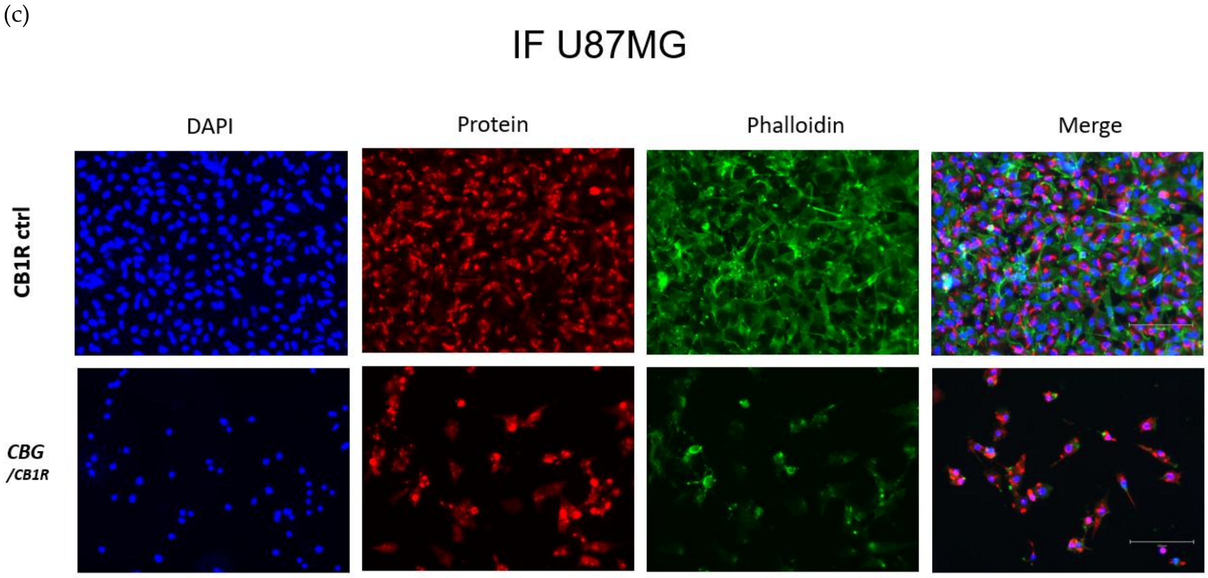

(d)

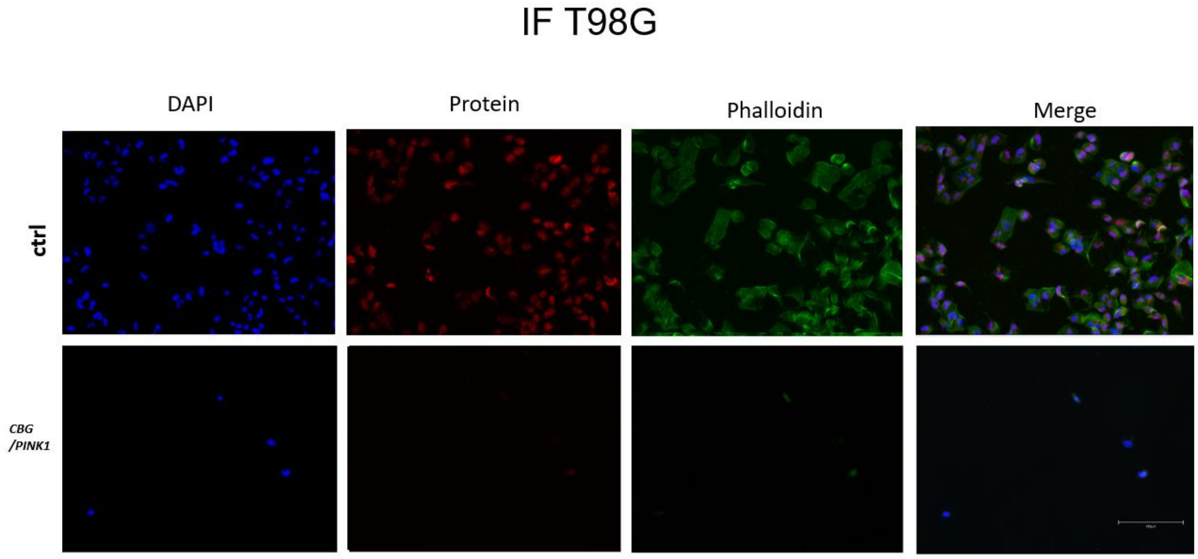

Figure S4. Immunofluorescence of PINK1, GPR55 and others in U87MG and T98G treated with control (Ctrl) and CBG 4 ng/μL for 24h and .analyzed via confocal microscopy (magnification ×20 scale bar 150 μm)

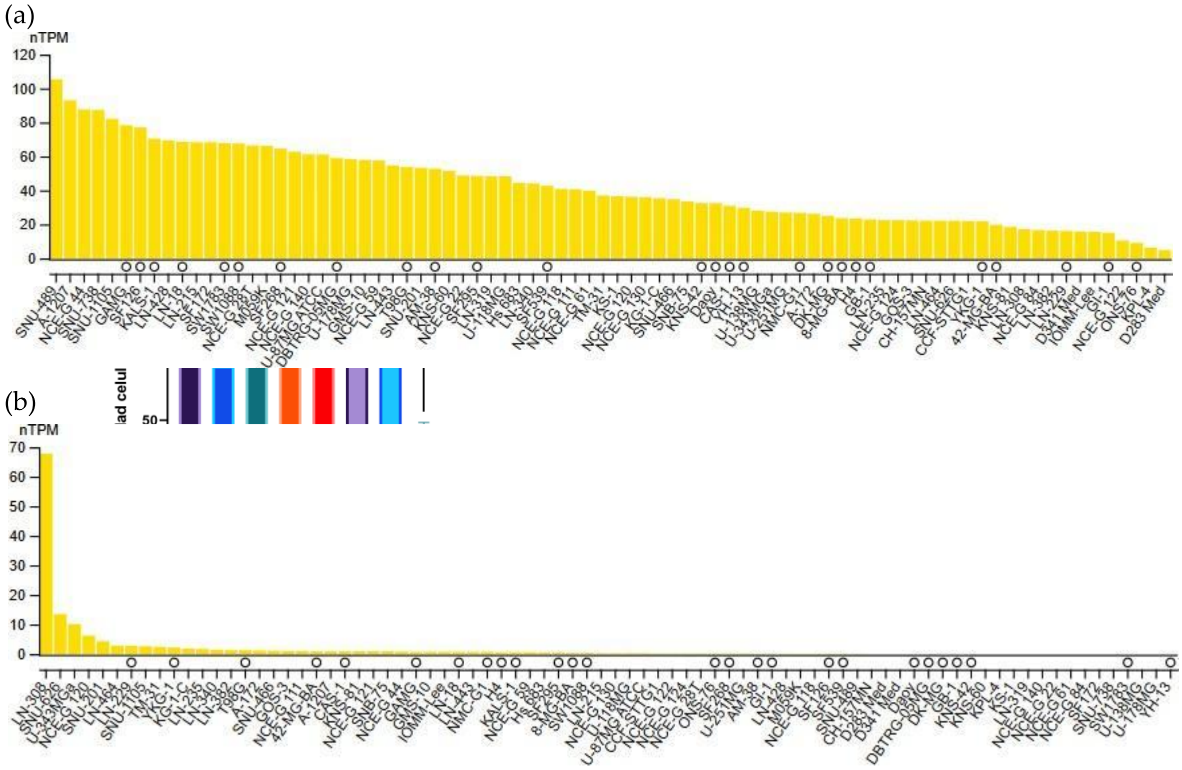

Figure S5. Expression level of (a) PINK1 and (b) GPR55 in different cell lines of brain tumors retrieved from RNA expression data as normalized transcript per million (nTPM) values of cancer cell lines. Similar to the cell line groups above, this plot show each individual nTPM of the gene in each cell line. More information and cell line data can be found in the Cell line resource. The analysis of the figures presented in this study highlights the interplay between cannabinoids and *Piper nigrum* derivatives in targeting glioblastoma multiforme (GBM). A observation from Figure 5 is the differential expression of PINK1 and GPR55 in various glioblastoma cell lines. Elevated GPR55 levels in glioblastoma cells, such as U87MG and CCF-STTG1, correlate with their invasive and proliferative capacities, emphasizing its role as a tumor-promoting factor. Conversely, the diminished expression of PINK1 in these cells may reflect compromised mitochondrial integrity and reduced mitophagic activity, further aligning with its reported role in tumor biology. These findings not only confirm previous observations but also strengthen the rationale for targeting these proteins therapeutically.

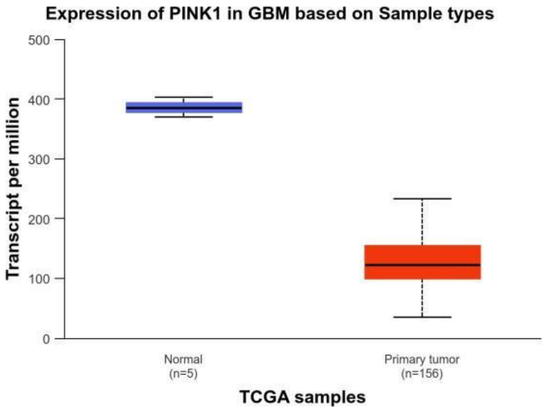

Comparison	Statistical significance
Normal-vs-Primary	1.11022302462516E-16

(a)

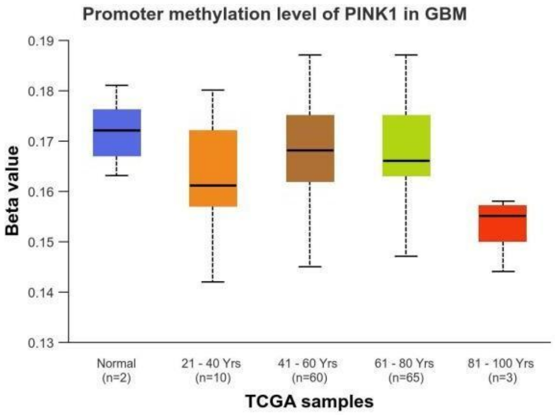

(b)

Age(41-60Yrs)-vs-Age(81-100Yrs)	3.655800E-02

Figure S6. Expression and promoter methylation level of PINK1. (**a**) The graph presents the expression of the PINK1 gene in normal tissue samples versus primary tumor tissue from patients with glioblastoma multiforme (GBM). It is observed that normal samples (in blue) show an average expression of approximately 400 transcripts per million, while primary tumor samples (in red) have a significantly lower expression, around 100 transcripts per million. The difference in expression is statistically significant, with a p-value of 1.11 x 10^-16, indicating that the reduction of PINK1 in primary tumors could have important implications for the understanding and treatment of GBM. (**b**) This image shows the promoter methylation levels of PINK1 in glioblastoma (GBM) samples across different age groups, compared to a normal control. The "Beta" values on the vertical axis represent the degree of methylation of the PINK1 promoter, and each age group of GBM patients is represented on the horizontal axis with a separate dataset.

In the context of glioblastoma, the methylation profile of PINK1 may have implications for the regulation of its expression. Generally, higher methylation of the promoter is associated with repression of gene expression, while lower methylation may allow for higher expression. From this image, it can be observed that the methylation level of PINK1 varies between age groups, being lower in individuals of older age (81-100 years) compared to younger age groups and the normal control group.

This pattern could suggest that the epigenetic regulation of PINK1 changes with age in GBM patients, which could have relevance for tumor progression or treatment response in these age groups. PINK1 is involved in mitochondrial homeostasis and the regulation of oxidative stress; therefore, its repression or activation through methylation may influence tumor behavior and cell survival in glioblastoma.

(a)

(b)

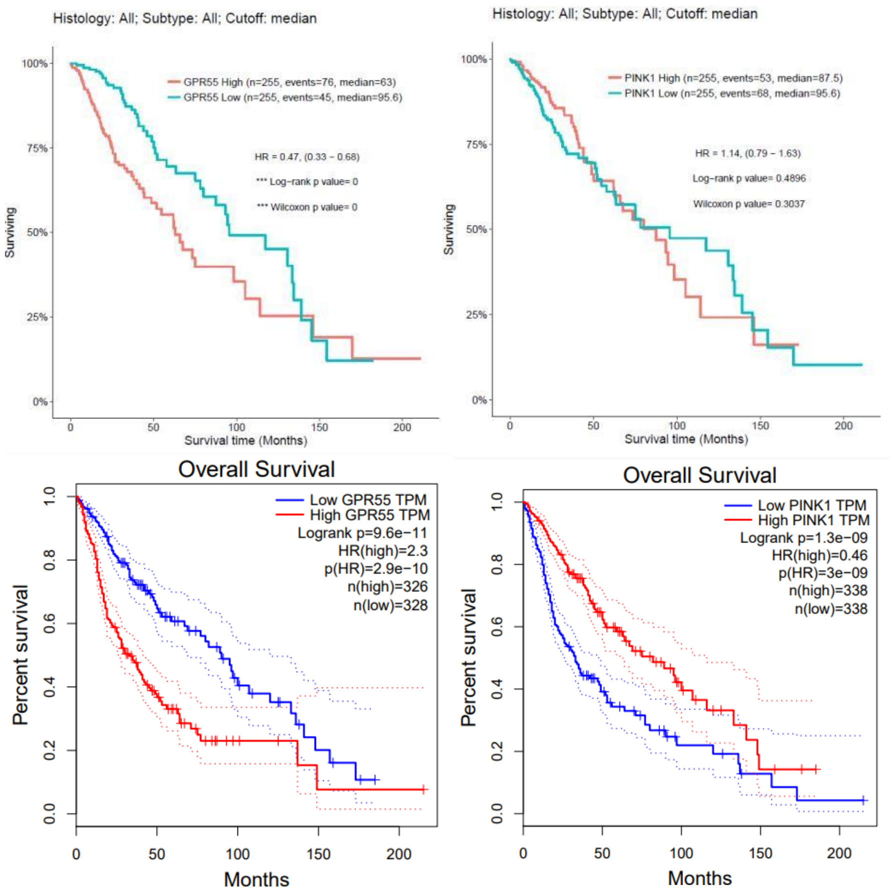

Figure S7. GBM vs LGG PINK1 and GPR55 expression data from (a) Gliovis and (b) GEPIA
